# Raindrops, Hailstones and Snowflakes

**Published:** 1881-12

**Authors:** 


### RAINDROPS, HAILSTONES AND SNOWFLAKES.

The usual Saturday evening lecture of the meeting of the British Association was delivered by Prof. Osborne Reynolds, who said that the subject of his lecture had not until recently been the subject of careful investigation, for certain explanations of these phenomena, dating very far back, and he might say founded on guess work, had never been accepted as satisfactory and sufficient. His object that night was to set before them the definite and rational history of the origin of these objects. He might call it the scientific history, for it was founded on the observations of physical laws, as well as tested by experiments which he hoped to put before them. The origin of his own special interest in this subject was the actual observance of what was open to every one. It was not till he was of mature age that he knew what really was the form of the ordinary hailstone, and this appeared to have escaped observation previously. He then explained that the form of the hailstone was that of an inverted umbrella, they were of a definite and exact form, in the shape of a cone having a rounded base and somewhat ribbed side. The lecturer showed a diagram illustrative of this description, and proceeded to explain the natural growth of these bodies. To apprehend that cloud or fog was formed by small definite particles or globules of water distributed through the air required no effort of the imagination. But when they came to inquire into the shape, size, and number of these particles, and as to their manner of suspension, there were certain difficulties before them. Let them start, for example, with the air in the hall in which they were assembled, presuming, of course, that it was clear air. In this air, strictly speaking, there was no water in the form of water, but there was water in the form of clear steam—gas as clear as air. The apprehension of this universal presence of steam in the lower strata of the atmosphere was the very foundation of the knowledge of meteorology. The amount of steam present depended almost entirely on the temperature. At the normal temperature of 60° Fahr., there were about six grains of steam in each cubic foot of air, or about a grain in a gallon. If the air were cooled to 30° there would only be present half as much steam, and the question was as to what would become of the other half. It would take the form of water which would be distributed through the room much as the steam was, but not exactly. The change from steam into water meant the congregation of the molecules into masses. It was obvious that a molecule of water at one end of the room could not get to the other end at once. What happened in the case of condensation of steam by the cooling of the air was very much the same as what would happen in the case of a regiment of infantry, in skirmishing order, being surprised by a regiment of cavalry. They would run into small knots in the best way they could. What happened when the air was cool was much the same. The particles which formed the water ran together into small groups, and formed those larger particles which constituted a fog. It was important to notice that there was a good physical reason which prohibited these elementary particles attaining more than a certain size. What they call the mean path of a molecule, or distance which a steam molecule was moving about practically limited the size of the water particle which was formed on the first condensation. These congregations of water-particles were for the most part extremely small. He then proceeded to explain what were the causes of cooling air, one of which was the air coming in contact and mixing up with colder air. This was the common cause of dew at night and very commonly of the clouds above. But the main cause of the cooling of the air was the result of what scientists called expansion, or, as he might describe it for his own purposes, the ascension of air from a position near the ground up to higher positions among the surrounding air. The summer storms, to which he called particular attention, as affording the best evidence of this phenomenon, might obviously be traced to the ascension of a column of air which had been warmed by the ground, ascending much in the form of a column of smoke, and as it rose and expanded it became cool. It was merely the ascension which brought about the cloud, and the conclusion was that if we had layers of color in the air so as to see what was going to happen, all those white flocky clouds would declare themselves to be nothing more than the higher points of a lower stratum of air. This was the case with summer storms in particular. In this way huge masses of cloud are formed. These clouds reached several miles in thickness. Indeed, we are apt to take a very poor idea of the size of clouds owing to their height and their distance. As to the particles of which he had been speaking, the first question was why they did not settle down. The fact was, however, that they did descend, but very slowly. He asked them to imagine a particle of two diameters descending, and then to imagine a particle of one diameter descending. They would both descend until the resistance of the air amounted to the same effect as their weight. That fact was pretty well known, but it was not until Prof. Stokes clearly showed that the resistance of the air was proportional to the velocity at which the particles were descending, that it was clear that the particles would descend with the velocities proportional to their diameters. The ordinary size of a raindrop was very small. If they imagine a particle the size of a raindrop, it descended with obvious velocity, but if they took a particle the size of a fog particle, if they diminished the velocity by one hundred, they would get a comparatively smaller velocity. He could not actually show the size of the particles—they were too small to put in his lantern, but he showed them as an analogous phenomenon, extremely small particles of air ascending in fluid. They were literally like particles of a fog descending. The point to which he next asked their attention was why those particles do not fall suddenly and completely down, or why they aggregated and formed a raindrop. The real point of his lecture had been to explain how it was that those separate particles which they saw in the clouds came together and formed raindrops. The size of a raindrop was usually over-estimated, but it was a large raindrop that was one-sixteenth of an inch in diameter, while an ordinary raindrop was not more than a thirty-second part of an inch in diameter. But as to the question how it was the particles in the clouds came together and formed a raindrop; the larger particles descended faster than the smaller particles, and, sweeping them up in their descent, fell to the earth as raindrops. That was the key to the whole subject. If it were not for the large particles descending faster than the others, the whole of the particles would descend like a regiment of soldiers, and though they would never form rain they would come to the earth in a dense and perpetual fog. The manner in which the particles became united was not so well ascertained; indeed, he did not know that electricity might not have something to do with it. This was one of the explanations why clouds sometimes rained and sometimes did not. Cloud particles falling through a dense cloud for 1,000 feet would acquire the size of one-sixteenth of an inch in diameter, which was the size of the largest raindrops. Enlarging further on the subject, the lecturer said that clouds were often four or five miles in thickness. He then lucidly spoke on the following topics: Why do we not have larger raindrops; why do drops take the form of spheres at all; and what keeps them in the form of drops? By experiments, he showed the accumulation of particles into a drop, and the falling of a shower of rain. Some of these experiments, he explained, had never been shown to a large public audience before. The next topic treated of was capillary attraction—that which held the drops together, and caused water to rise up in small spaces. This showed conclusively that the surface of water was of a certain definite tension. The lecturer then proceeded to explain that when a raindrop exceeded a certain size its tension was insufficient to sustain it, and it fell and burst by virtue of the pressure against it. If it were not for this they would have drops amounting in size to the larger hailstones which sometimes fell. It would be a new phenomenon to have raindrops an inch or two inches in diameter, and what would be the result if it did happen might better be imagined than described. The fact that ice particles would stick together was not at first obvious; but a little consideration at once showed that that must be. With regard to the shape of hailstones, he said the conclusion generally come to was that that form was the result of the breaking up of a larger spherical stone having a vertex of the cone in its center.

